# Risk of upper limb complaints due to computer use in older persons: a randomized study

**DOI:** 10.1186/1471-2318-7-21

**Published:** 2007-08-16

**Authors:** Martin PJ van Boxtel, Karin Slegers, Jelle Jolles, Joop M Ruijgrok

**Affiliations:** 1 European Graduate School of Neuroscience (Euron); Brain and Behaviour Institute, Maastricht University, Department of Psychiatry and Neuropsychology, PO Box 616, 6200 MD Maastricht, The Netherlands; 2 Vodafone, Group R&D, Gelissendomein 5, 6229 GK Maastricht, The Netherlands; 3 Department of Rehabilitation, Maastricht University Hospital, PO Box 5800, 6202 AZ, Maastricht, The Netherlands

## Abstract

**Background:**

We studied whether the twelve-month use of a standard computer would induce complaints of upper limb pain or functional limitations in older novice computer users.

**Methods:**

Participants between 64 and 76 of age were randomly assigned to an Intervention group (n = 62), whose members received a personal computer and fast Internet access at their homes, or a No Intervention control group (n = 61), whose members refrained from computer use during the twelve month study period.

**Results:**

Difference scores between baseline and twelve months assessments on both complaint (SFS) and functional health scales (SF-36) did not differ between groups (all *p *> .05).

**Conclusion:**

Prolonged, self-paced use of a standard computer interface does not put older persons at a risk of upper limb complaints or reduce functional health in older adults.

## Background

The personal computer has become an ubiquitous technology, both at home and in the workplace. Everyday computer activities are generally characterized by repetitive upper limb movements and a relatively fixed bodily position [1]. Regular interaction with a computer interface, using a standard keyboard and a mouse, has been related to a complex of complaints related to the hand, arm and shoulder, often referred to as 'repetitive strain injury' (RSI), or sometimes as 'cumulative trauma disorder', 'non-specific work-related upper limb disorder' or 'repetitive strain disorder' [2]. This pain syndrome of the upper limb consists of protracted complaints of the hand, arm or shoulder, leading to functional impairment that is difficult to treat [3]. Although the exact cause of RSI is still unknown, risk factors have been identified in epidemiological studies. Prolonged exposure to repetitive movements and less than optimal ergonomic conditions have been shown to increase risk, while secondary factors like working under time pressure and lack of autonomy and social support in the workplace can increase the frequency and severity of complaints [3]. Little is known, however, about the relative contribution of each risk factor and any possible synergistic effect.

Exact prevalence figures of RSI are unavailable, in part due to a lack of valid working definitions of RSI as a diagnostic entity, but in a recent Dutch population survey RSI complaints were reported by 20–40 percent of the working population [4]. So far, all studies done in this area have been observational in nature and were restricted to studies in the workplace in young to middle-aged persons. In non-working older computer users even prevalence figures about RSI are lacking.

More insight into the long-term effects of computer use may be of particular relevance for older individuals because they may be even more susceptible to the development of RSI related complaints compared to younger or middle-aged persons. Firstly, hand function deteriorates with age due to age-related degenerative changes in the musculoskeletal, nervous and vascular system [5]. Besides these more general degenerative mechanisms, hand function in the elderly can be impaired by specific pathological conditions, such as osteoporosis, osteoarthritis, or rheumatic arthritis [5,6]. Secondly, recent insights into neural control of movements and muscle stiffness regulation provide further arguments for the older population being more at risk. The neuromotor noise theory explains why fine, coordinated distal movements require strong signals from the brain to the muscular system [7]. This signal can be disturbed by different types of task-irrelevant neural activity. Such neural 'noise' can be generated by different sources, e.g. sensory input, parallel activity in adjacent cognitive networks ('double tasks'), or mood related brain centres [8]. In order to improve the signal-to-noise ratio, the brain will suppress excess noise by stiffening the muscular system. For instance, it has been shown that increasing time pressure and task difficulty will cause enhancement of muscular tone [9]. It can be argued that an older brain will produce a less strong motor signal, is less able to suppress neural noise, and will intrinsically produce more noise [10]. Finally, evidence of age-related changes that may cause an unfavourable signal-to-noise ratio in the brain, thereby decreasing hand function, also comes from patient studies, e.g. into multiple sclerosis [7] and stroke [11].

Thus, while individuals over 65 have been catching up fast with the societal trend towards extensive use of computer-based technologies and services [12], it remains unclear whether computer usage may put the older users to the risk of RSI. Even though the nature and intensity of computer use may differ between professionally active younger persons and non-working older individuals, the susceptibility of the latter group to develop RSI may be higher due to the mechanisms outlined above. The present prospective study investigates if older computer users actually develop complaints or functional limitations of the upper limb in the course of a one-year computer and Internet training program. The study was part of a larger project into the effects of computer use on cognitive abilities and life quality in older adults [14]. All participants who were trained for the actual intervention study were enrolled in the current study. They were interested in computer use for leisure and educational purposes, but had no prior computer experience. Apart from the use of a specific RSI questionnaire, measures of general health were included in this study because upper limb bodily pain may be related to a reduction in overall health status in both younger and older individuals [13].

## Methods

### Participants and Procedure

Participants in the main project were community-dwelling older individuals aged between 64 and 76, both with and without interest in computer usage. They were randomly recruited by direct mail using address information from the electoral roll of the city of Maastricht, the Netherlands. The project flyer contained information about the main project, which aimed at investigating the effect of twelve month exposure to computer and Internet usage on cognitive abilities and wellbeing [14]. Individuals with no prior computer experience, again both with and without interest in learning to master computer skills for personal use, were invited to request more information about the study by returning a prepaid postcard. Apart from a dementia screening at baseline (score on the Mini-Mental State Examination of 24 or higher [15]), the ability to travel to the research centre, and agreement to refrain from computer usage for the duration of the study in the control groups, there were no inclusion criteria for the main project. Participants in the main study without interest (n = 45) were not included in the study. Participants with interest in using a computer (n = 191) were randomly assigned to three groups in a two-phased randomization procedure. Two-thirds of the participants with interest were randomly selected for a three-session training course in general computer and Internet skills (n = 123). The remaining participants in the main study with interest but who were not trained (n = 68) were also not included in the present study. During the training participants were instructed in three two-hour sessions on how to use the computer and common software applications, such as an Internet browser, email and a word processor. After the training, the 123 participants were randomly assigned to an 'Intervention' group (n = 62) and a 'No Intervention' group (n = 61). As stated above, only these two groups were part of the current study, as they differed only with respect to the intervention proper. Participants returned for follow-up assessments after four and twelve months. Data on outcome variables for this study were obtained at baseline (M0) and the 12 month follow-up (M12). Follow-up data were available for 60 and 49 participants in the intervention and control group, respectively. Data for fourteen participants were not available for several reasons: health problems (n = 2), time constraints (n = 2), private problems (n = 2), disappointment in randomization results (n = 2), death of partner (n = 1), moving (n = 1), or no clear reason (n = 1). Furthermore, two participants could not be reached for an appointment after several attempts, and one participant died. Five participants who completed the test sessions did not return their questionnaire even after repeated requests, leaving 57 and 47 participants in the Intervention and No Intervention groups with complete data.

The No Intervention group was to refrain from computer use during the intervention interval of twelve months, which was confirmed in writing on the informed consent form. Compliance with this agreement was again confirmed by signing a statement at the end of the study. Participants in the No Intervention group could win one of two computer systems in a raffle at the end of the study period. Participants in the Intervention group were equipped with an up-to-date personal computer (Apple iMac) with high-speed Internet access (cable). A standard QWERTY keyboard and a one-button mouse were used as input devices. Regular home assignments were given by email to ascertain continuous use of the computer facilities and to track down participants who made insufficient progress with respect to their computer skills. A help desk with remote support facilities was available for all questions related to computer and Internet use during the project. All participants signed a consent form, and the study was approved by the ethics committee of the Maastricht University Hospital (reference number MEC01-116.3).

### Measurements

A standard questionnaire was completed at the start (M0) and the end (M12) of the study. It contained questions about demographical characteristics (e.g. educational level, marital status), health status, (pain) medication use, and psychological and general wellbeing. At M12 information was collected about the total amount of time (hours per week) devoted to computer and Internet usage. A standard battery of cognitive tests was given at M0 and M12 to assess cognitive ability used in the main study. Results of this assessment will be described in an upcoming paper by these authors.

### Specific measures

The Short Form-36 (SF-36) scale [16] was used at both M0 and M12 to assess the general wellbeing of the participants. To restrict the number of outcome variables in order to prevent type II error, we a priori selected only the most relevant subscales from the SF-36 for this study: general health, physical functioning, mental health and bodily pain. All scores refer to health complaints that were experienced in the four weeks preceding the testing. Scores on the SF-36 scales range between 100 (highest; optimal) and 0 (lowest; worst).

Complaints about and functional impairment of the upper limb were measured with the symptom and functional status scale (SFS), a well-validated instrument that was originally developed for the assessment of upper limb pain, specifically the carpal tunnel syndrome [17]. It consists of eleven symptom items (e.g. 'How long, on average, does an episode of pain last during the daytime?') and eight functional impairment items (e.g. difficulties with writing), measured on five-point scales, with reference to the two weeks prior to testing. Domain scores for symptom severity and functional status were computed by taking the average of the items in each category. Thus the range of scores was between 1 (no complaint/impairment) to 5 (maximum severity of complaint/impairment).

### Statistical analysis

All outcome measures failed a formal test of normality (Kolmogorov-Smirnov test), due to skewed distributions. M12-M0 difference scores were calculated for all SF-36 and SFS scales, which were tested for group differences with Mann-Whitney *U *tests. All analyses were conducted according to the intention to treat principle. A post hoc power analysis using an alpha level of .05 revealed that a power of 0.81 was available to detect 'medium effect size' differences based on the number of participants who completed all assessments [18]. All analyses were performed with the SPSS Program Series, v11.02 for Apple Macintosh, using an alpha level of .05 for significance and of .10–.05 for marginal significant effects.

## Results

Table 1 presents the baseline characteristics of both study groups. At the twelve-month follow-up moment, participants in the Intervention group reported an average computer use of 8.3 (SD = 6.2) hours per week. In total 6.5 hours (SD = 5.6) per week was spent on Internet-related activities (web surfing, e-mail). Incidental or regular pain medication use did not differ between groups before and after the intervention (M0: 16 versus 21 percent; M12: 19 versus 19 percent in the intervention and control group, respectively, *Chi-square p *> .05).

**Table 1:** Descriptive data for demographical variables and outcome measures at M0 and M12 in both study groups. The male/female ratio was 25/32 and 21/26 in the Intervention and No Intervention groups, respectively.

	Intervention		No Intervention	
	Mean	SD	Mean	SD
Age (years)	69.0	2.7	69.1	2.8
Educational Level	3.7	1.5	3.8	1.8
				
	Median	P25-P75	Median	P25-P75
SF-36: General Health M0	70	60–80	75	65–80
SF-36: General Health M12	70	60–80	65	60–75
SF-36: Physical Functioning M0	90	80–95	90	80–95
SF-36: Physical Functioning M12	85	80–95	90	75–95
SF-36: Mental Health M0	84	72–92	84	72–88
SF-36: Mental Health M12	84	72–88	84	76–88
SF-36: Bodily Pain M0	100	79–100	100	90–100
SF-36: Bodily Pain M12	90	67–100	90	67–100
SFS: Functional Status M0	1.00	1.00–1.06	1.00	1.00–1.25
SFS: Functional Status M12	1.00	1.00–1.13	1.00	1.00–1.13
SFS: Symptom Severity M0	1.00	1.00–1.18	1.00	1.00–1.18
SFS: Symptom Severity M12	1.00	1.00–1.09	1.00	1.00–1.18

SF-36 = Short Form-36 scale; SFS = symptom and functional status scale.

P25-P75 = 25–75^th ^percentile range.

When both groups were compared with respect to pre- to post-intervention changes on health and complaint scales, no differences were observed (all *Z *> -1.94, *p *> .05), indicating that there was no intervention-related differential change over time in the groups with respect to general health, functional status or upper limb complaints (Table 2). Two scores approached a significant difference in both groups. The scores on the SF-36: General Health were marginally lower at M12 in the No Intervention group when compared to the Intervention group (*p *= .079). The SFS: Functional Status scores showed a small reduction in complaint level in the No Intervention group only (*p *= .052).

**Table 2:** M12 (12 month follow-up) minus M0 (baseline) difference scores on all outcome variables in both study groups. Between group differences were tested with Mann-Whitney *U *tests.

	Intervention		No Intervention		
	Median	P25-P75	Median	P25-P75	*p*
SF-36: General Health M12-M0	.00	-10.00–2.50	-5.00	-15.00–0.00	.079
SF-36: Physical Functioning M12-M0	.00	-5.00–2.50	.00	-10.00–5.00	.797
SF-36: Mental Health M12-M0	.00	-8.00–6.00	.00	-4.00–4.00	.569
SF-36: Bodily Pain M12-M0	.00	-20.41–0.00	.00	-22.45–0.00	.642
SFS: Functional Status M12-M0	.00	0.00–0.03	.00	-0.13–0.00	.052
SFS: Symptom Severity M12-M0	.00	0.00–0.00	.00	-0.09–0.00	.878

SF-36 = Short Form-36 scale; SFS = symptom and functional status scale.

P25-P75 = 25–75^th ^percentile range.

## Discussion

In this study we tested whether the prolonged use of a standard computer interface puts older novice users at risk of poorer functional health and upper extremity complaints. This is the first randomized study into the effects of computer and Internet use on long-term functional status. No clear indication was found that participants who were part of the Intervention group were at greater risk as regards the development of health complaints or functional impairment of the upper limb than participants in the control group. Still, although the general health level marginally decreased in the No Intervention group, the small reduction in SFS: Functional Status complaint level in this group was not present in the Intervention group. The latter finding may be due to random variation but could be indicative of a trend towards an unfavourable effect in the Intervention group. This would become more likely if a tendency towards lower scores on the SFS scale after repeated administration was documented with this scale in people with no complaints. To our knowledge, however, such trend has not been reported in the literature to date, so we do tend to ascribe these marginally significant findings to random variation. Furthermore, both marginally significant differences would become insignificant when a Bonferroni correction for multiple testing would be applied: after dividing the alpha range by the number of tests (6), the range to render a test marginally significant would become .016 to .008.

The absence of differences between the two groups in reported health complaints at M12 may be a reflection of the lack of sufficient risk factors relating to developing RSI-like symptoms. Firstly, although the participants were well-motivated computer users, the mean time spent on computer related activities in the Intervention group was limited to 8.3 hours. This is lower than the exposure of professional workers who use computers on a daily basis [20] but is comparable to the average use in this age group of 7.7 hours (2002–2004) reported in a recent Dutch survey [12]. Secondly, it has been found that adverse psychological factors, including work-related stress, may add to the effect of repetitive movements of the arms and wrists in the etiology of upper limb complaints [21,22]. Since our participants used the computer mainly for personal goals, this is another reason why the study group may have a lower risk of developing RSI-like complaints than those who are professionally active computer users. Thirdly, unfavourable ergonomic conditions, such as prolonged fixed body postures, which are common in working environments in the presence of time pressure, are also less likely to occur in a home situation. The question remains as to whether older users who engage more intensively in computer-related activities, or who continue such activities for a longer period of time, may develop upper limb complaints at a later stage.

There are some methodological limitations in this study. First, we did not choose to do a specific diagnostic workup to detect functional impairments of the upper limb in accordance with clinical standards. No comprehensive assessment protocol is available to reliably test functional impairments of the forearm in the near normal range. Furthermore, application of a functional assessment test was considered to be prone to measurement bias as it would be difficult to test participants unaware of the actual rationale behind such tests. The SFS scales to quantify upper limb pain was developed particularly to measure pain complaints in the hand and wrist regions but may be less sensitive to quantify complaints in the higher regions of the upper extremity, like shoulder and elbow. At the exit interview no participant in the intervention group expressed such complaints. Next, we chose to use selfreport measure of actual computer usage. Our estimate of computer usage was derived from earlier population surveys on computer usage in the workplace, for which no data on reliability are available. Self-report measures may be sensitive to recall bias or social desirability and therefore are sometimes considered less accurate than objective measures. Other more objective methods have been considered (e.g. logon time, keystroke logging), but were discarded because they were potentially restrictive to the user, had other intrinsic flaws (e.g. uncertainty about user identity) and application without prior notification would be unethical. However, it was made quite clear to all participants that they could use the computer at there own discretion and their own pace without risk of disapproval by the researchers in case their motivation was low, which may have reduced the chance of an overestimation of actual computer use by the participants. Finally, no systematic inventory was made of individual risk factors, such as ergonomics of the work place, subjective psychological distress, or underlying health disorders causing impairment of hand function.

## Conclusion

In summary, older users of a standard computer interface with no prior computer experience did not appear to be at greater risk of a poorer general health or of more symptoms or functional impairment of the upper extremity after a twelve-month episode of average, selfpaced use. Any reserve to computer use in older persons prompted by putative negative effects on the musculoskeletal system is therefore not supported by our empirical evidence. However, when risk factors are apparent (e.g. osteoarthritis), special precautions may still be necessary in individual cases to prevent symptoms at a later stage, e.g. by maintaining an ergonomic posture, or by avoiding prolonged computer usage.

## Abbreviations

RSI, Repetitive Strain Injury; SF-36, Short Form 36 questionnaire; SFS, Symptom and Functional Status scale.

## Competing interests

The author(s) declare that they have no competing interests.

## Authors' contributions

MvB conceived of the study, executed the data analysis and prepared the first manuscript draft. KS carried out the actual trial and assisted in the data analysis. JJ and JR took part in the design phase of the study and JR provided the rationale for RSI assessment. All authors have read and approved the final version of the manuscript.

## Pre-publication history

The pre-publication history for this paper can be accessed here:


